# The Interplay Between Autophagy and Porcine Epidemic Diarrhea Virus: From Molecular Mechanisms to Therapeutic Perspectives

**DOI:** 10.3390/microorganisms14091890

**Published:** 2026-08-26

**Authors:** Zhihua Feng, Cunyi Qiu, Zhiding Zhou, Meilin Yang, Huaxin Wang, Yefei Zhou

**Affiliations:** 1 College of Veterinary Science and Technology, Gansu Polytechnic College of Animal Husbandry & Engineering, Wuwei 733006, Chinavetqcy5495@nwafu.edu.cn (C.Q.);; 2 Department of Food Science, Nanjing Xiaozhuang University, Nanjing 211171, China; 3 College of Veterinary Medicine, Sichuan Agricultural University, Chengdu 611130, China

**Keywords:** autophagy, porcine epidemic diarrhea virus, mitophagy, innate immunity, endoplasmic reticulum stress, antiviral strategy

## Abstract

Autophagy is a highly conserved degradation and recycling process in eukaryotic cells that plays a critical role in maintaining cellular homeostasis and responding to external stress. During viral infection, autophagy exhibits a classic “double-edged sword” effect—it can act as a host defense mechanism by directly degrading viral components, but it can also be hijacked by viruses to promote their own replication. Porcine epidemic diarrhea virus (PEDV), an important enteric coronavirus that severely affects the global swine industry, engages in a complex and sophisticated interplay with the host autophagy system. This review systematically dissects the dual regulatory mechanisms of autophagy during PEDV infection and reveals two intertwined functional axes. On one hand, PEDV utilizes multiple viral proteins to cooperatively manipulate the autophagic pathway—inducing mitophagy to suppress innate immune responses, utilizing autophagic membranes to construct replication platforms, and blocking autophagic flux to evade degradation—thereby establishing a multi-level pro-viral network. On the other hand, host cells deploy a unified molecular axis of “ubiquitination–autophagy receptor–lysosome” by mobilizing a broad array of restriction factors to target and degrade viral proteins, forming a coordinated defense system. These two axes converge at the oxidative stress–endoplasmic reticulum stress–autophagy hub, where PEDV NSP1 and NSP2 synergistically inhibit the NRF2 antioxidant system to trigger this cascade, while host factors such as DDX6 and ACE2 finely regulate the process. Based on this mechanistic framework, we discuss the therapeutic implications of targeting autophagy for PEDV intervention, with particular emphasis on the development of selective autophagy modulators as potential antiviral agents. We also identify key knowledge gaps and propose future research directions to translate these mechanistic insights into clinical or field applications. This review synthesizes the peer-reviewed literature published between 2013 and 2026, identified through systematic searches of PubMed, Web of Science, and Scopus databases. Notably, the majority of mechanistic findings discussed are derived from in vitro cell culture models, and their translation to in vivo settings remains a significant challenge. Bridging this gap will require validation in physiologically relevant models, such as porcine intestinal organoids and controlled piglet challenge studies, to assess the efficacy and safety of autophagy-targeting interventions in the context of intestinal homeostasis and mucosal immunity.

## 1. Introduction

Autophagy is a highly conserved degradation and recycling process in eukaryotic cells, first described in 1963 by Belgian scientist Christian de Duve [1]. Its core function is to form double-membrane autophagosomes that engulf damaged organelles, misfolded proteins, or invading pathogens [2] and deliver them to lysosomes for degradation. The degradation products—including amino acids, fatty acids, and nucleotides—are then recycled to maintain intracellular homeostasis and to cope with external stressors such as nutrient deprivation and oxidative stress [3,4]. Based on the route of substrate delivery to lysosomes, autophagy can be classified into three types: macroautophagy, microautophagy, and chaperone-mediated autophagy (CMA) [5]. Based on substrate specificity, it can be divided into non-selective and selective autophagy. Selective autophagy uses cargo receptors (e.g., p62, NBR1, NDP52, Tollip) to precisely recognize ubiquitin-tagged specific substrates [6], enabling fine-tuned cellular regulation and playing a key role in antiviral immunity. Together, these three types form a diversified regulatory network that operates coordinately under various physiological and pathological conditions.

The interaction between autophagy and viruses exhibits a classic “double-edged sword” effect, with this duality dynamically changing at different stages of infection and in different cellular environments [7,8]. On the antiviral side, autophagy can directly recognize and degrade invading viral particles or viral proteins through xenophagy. For example, the capsid protein of chikungunya virus and the VP1 protein of foot-and-mouth disease virus are recognized by the autophagy receptors p62 and NDP52 and degraded via the lysosomal pathway [9]. Autophagy can also reduce reactive oxygen species (ROS) accumulation by clearing damaged mitochondria [10], and activate signaling pathways such as cGAS-STING to promote interferon production and antigen presentation [11], thereby enhancing innate and adaptive immune responses. On the pro-viral side, many viruses have evolved sophisticated evasion or exploitation strategies during long-term co-evolution. Some viruses (e.g., hepatitis C virus) use autophagic membranes as “replication factories” [12], hijacking autophagy-related proteins to induce autophagosome accumulation while blocking autophagosome–lysosome fusion to create favorable conditions for viral replication and release. Others (e.g., herpes simplex virus type 1) encode specific proteins (such as ICP34.5) that inhibit autophagy initiation or degrade key autophagic factors, thereby escaping autophagic clearance [13]. This complex relationship—in which autophagy can either exert antiviral effects or be exploited for viral replication—makes it a key regulatory node in viral infection research.

**Figure 1:**
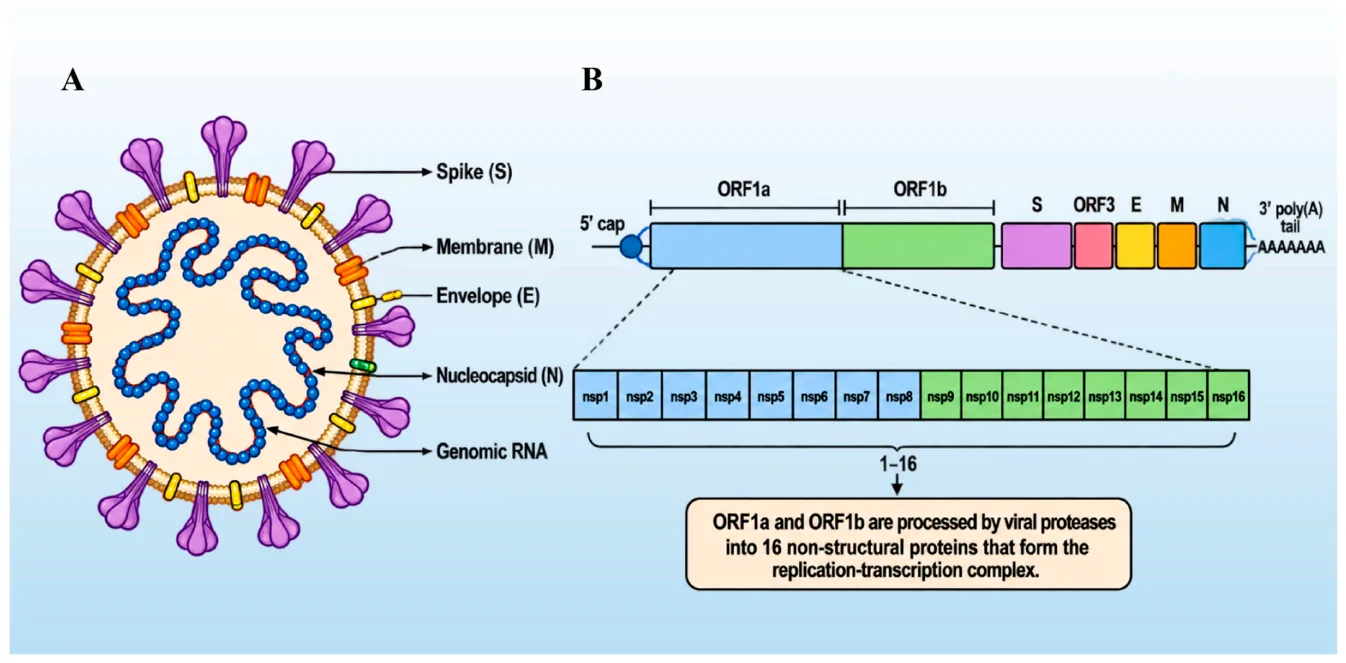
Schematic illustration of PEDV virion structure and genome organization. (**A**) Virion structure showing the envelope (E), membrane (M), spike (S) glycoprotein projections, and nucleocapsid (N) protein encapsidating the genomic RNA. (**B**) Genome organization showing the 5′ cap, 3′ poly(A) tail, and open reading frames (ORFs) encoding non-structural proteins (nsp1–nsp16), structural proteins (S, E, M, N), and the accessory protein ORF3. The large ORF1a and ORF1b are processed by viral proteases into 16 non-structural proteins that form the replication–transcription complex. Not drawn to scale.

PEDV is a member of the genus *Alphacoronavirus* in the family *Coronaviridae*. It possesses a positive-sense, single-stranded RNA genome of approximately 28 kb, organized into at least seven open reading frames (ORFs) arranged as 5′-ORF1a/1b–S–ORF3–E–M–N-3′ [14,15,16]. The large ORF1a and ORF1b encode two polyproteins that are processed into 16 non-structural proteins (nsp1–nsp16) [17], which assemble into the viral replication–transcription complex responsible for genome replication and immune evasion [18]. The remaining ORFs encode four structural proteins—spike (S), envelope (E), membrane (M), and nucleocapsid (N)—and an accessory protein ORF3 (Figure 1). Among these, the S protein is a major target of neutralizing antibodies and is one of the most heavily glycosylated proteins known, features that have profound implications for immune pressure and vaccine development. PEDV infection causes acute watery diarrhea, vomiting, dehydration, and anorexia in piglets, with mortality rates reaching up to 100% in neonatal piglets and 90–95% in suckling piglets, while dropping to less than 10% in piglets older than 10 days [19,20]. The virus primarily targets the villous intestinal epithelium of the small intestine, leading to villous atrophy, disruption of tight junctions, and malabsorption [21]. PEDV is endemic in major pork-producing regions worldwide, including Asia, North America, and Europe [22]. Phylogenetically, strains are classified into genogroup 1 (G1, classical strains) and genogroup 2 (G2, pandemic strains), with G2b being the predominant lineage circulating globally [23]. Genomic analyses of 672 PEDV strains have identified the fast-evolving G2 strains as the predominant epidemic viruses, with the G2c sub-genotype accounting for over 70% of circulating strains in China [16]. The economic impact has been devastating—PEDV caused losses exceeding approximately USD 1 billion during the 2013–2014 outbreaks alone [24]. The viral replication cycle begins with S protein binding to the host receptor aminopeptidase N, followed by entry via clathrin-mediated endocytosis, cytoplasmic genome replication in association with double-membrane vesicles, and release of progeny virions through the secretory pathway [25]. Despite more than four decades of research, effective control of PEDV remains elusive. Current prevention and control strategies face two major challenges. First, most inactivated and attenuated vaccines are administered intramuscularly, which fails to effectively activate the gut–mammary gland–sIgA axis, leaving piglets without sufficient protective sIgA antibodies from colostrum [26]. Second, the S protein—under constant immune pressure—frequently undergoes mutations, and amino acid changes in key neutralizing epitopes often lead to vaccine failure [27,28]. These challenges underscore the urgent need to elucidate the molecular interplay between PEDV and host cells, particularly the multiple roles of autophagy in this process, as a basis for developing novel antiviral strategies.

Despite more than four decades of research, PEDV remains endemic in many parts of the world. In recent years, substantial progress has been made in understanding the interaction between autophagy and PEDV [29,30]. While existing reviews have summarized PEDV immune evasion mechanisms [31] or host restriction factors, a comprehensive synthesis that integrates both the pro-viral and anti-viral roles of autophagy within a unified mechanistic framework has been lacking. The literature presents seemingly contradictory findings on whether autophagy promotes or suppresses PEDV replication. We propose that these discrepancies can be reconciled by examining the integrity of autophagic flux: PEDV infection establishes an “incomplete autophagy” state characterized by autophagosome accumulation but impaired lysosomal fusion. Interventions that exacerbate this blockade promote viral replication, whereas those that restore flux completion suppress it. This flux-centric framework, together with the molecular axes described above, serves as the organizing principle of this review. The review aims to move beyond descriptive enumeration of phenomena to reveal the intrinsic logic of the interplay between autophagy and PEDV, and to explore the potential of targeting this interplay for antiviral intervention, thereby providing new theoretical foundations and research perspectives for the control of PEDV.

## 2. Literature Search Strategy

This review was conducted following the preferred reporting items for systematic reviews and meta-analyses (PRISMA) guidelines where applicable. We performed systematic searches of PubMed, Web of Science, and Scopus databases for the peer-reviewed literature published between January 2013 and June 2026. The search terms included combinations of the following keywords: “porcine epidemic diarrhea virus,” “PEDV,” “autophagy,” “mitophagy,” “autophagic flux,” “selective autophagy,” “autophagy receptor,” “ubiquitination,” “ER stress,” “endoplasmic reticulum stress,” “innate immunity,” and “antiviral.” Boolean operators (AND, OR) were used to combine search terms. In addition, the reference lists of retrieved articles and relevant review papers were manually screened to identify any additional publications not captured by the database searches.

Inclusion criteria were: (i) original research articles, reviews, and short communications; (ii) studies investigating the interplay between autophagy and PEDV or related coronaviruses; and (iii) articles published in English. Exclusion criteria were: (i) conference abstracts, preprints, and non-English articles; (ii) studies not directly related to autophagy or PEDV; and (iii) duplicate publications.

A total of 847 records were initially identified. After removing 203 duplicates, 644 records were screened by title and abstract. Of these, 168 were excluded as irrelevant to the scope of this review, leaving 476 full-text articles assessed for eligibility. Following detailed evaluation against the inclusion and exclusion criteria, 312 articles were ultimately included in this review. Based on the reference list of the revised manuscript, 120 peer-reviewed articles are cited in this review. The literature search and screening process were performed independently by two authors (Z.F. and C.Q.), with disagreements resolved through discussion with a third author (Y.Z.).

## 3. Molecular Mechanisms by Which Autophagy Promotes PEDV Replication: A Multi-Level Manipulation Strategy of the Virus

**Figure 2:**
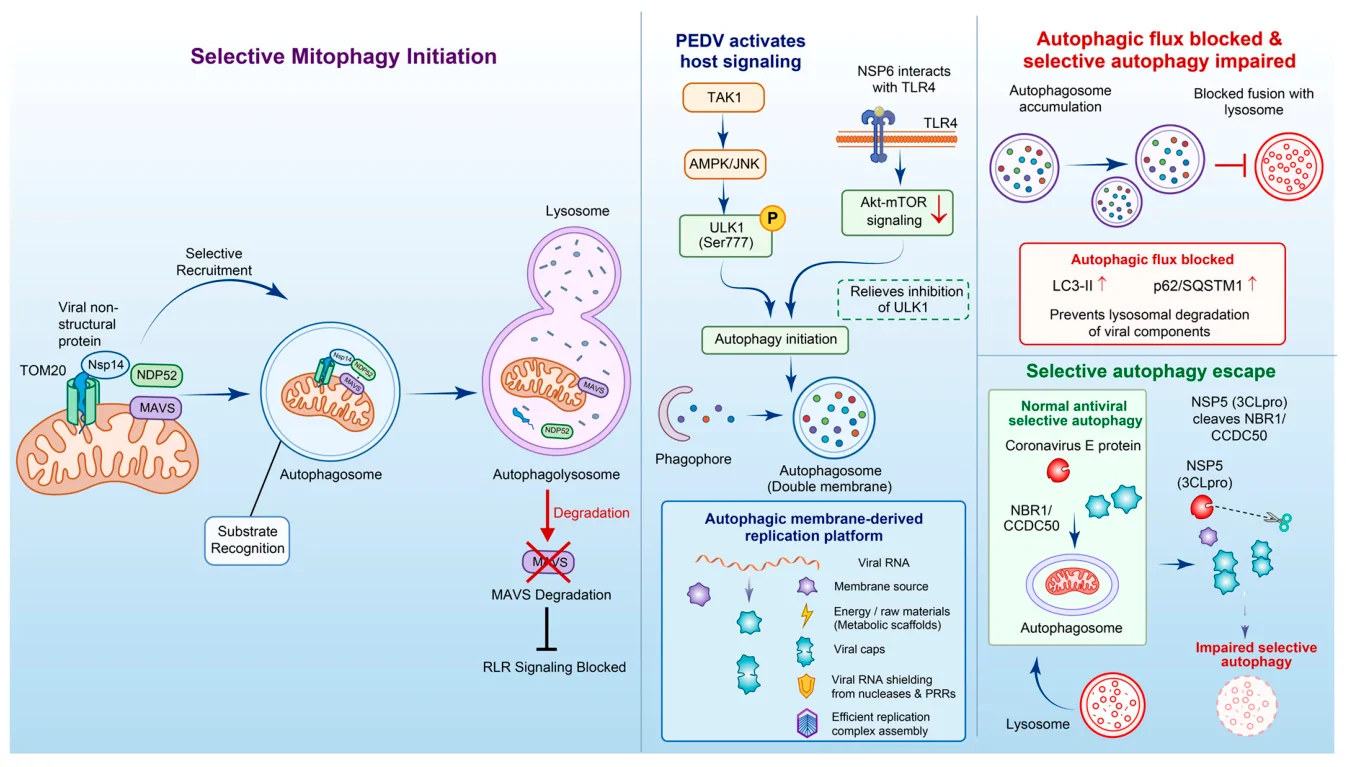
PEDV manipulates host autophagy at multiple levels to establish a pro-viral environment. Viral Nsp14 recruits the selective autophagy receptor NDP52 via TOM20 to induce mitophagy, resulting in MAVS degradation and suppression of RLR-mediated innate immunity. Concurrently, PEDV promotes autophagosome formation through two pathways: TAK1–AMPK/JNK–ULK1 signaling and NSP6–TLR4-mediated inhibition of Akt–mTOR, relieving ULK1 repression. The accumulated autophagosomes provide membrane scaffolds and metabolic resources for viral replication complexes. PEDV also impairs autophagosome–lysosome fusion, as indicated by elevated LC3-II and p62/SQSTM1 levels, thereby preventing lysosomal degradation of viral components. Additionally, although host cells can target the viral E protein via selective autophagy receptors NBR1 and CCDC50, PEDV counteracts this by using NSP5 (3CLpro) to cleave these receptors. Thus, PEDV converts host autophagy from an antiviral defense into a system that suppresses innate immunity, supplies replication membranes, and evades autophagic clearance.

During long term evolution, PEDV has developed a sophisticated strategy to manipulate the host autophagy system at multiple levels through the cooperative action of various viral proteins, serving its own replication needs [32] (Figure 2). These strategies do not act in isolation but are interwoven and progressive, collectively forming a comprehensive pro-viral network. This section summarizes the mechanisms by which PEDV manipulates autophagy to promote viral replication—collectively referred to as proviral autophagy—in contrast to the antiviral autophagy discussed in Section 4.

### 3.1. Hijacking Mitophagy to Suppress Innate Immunity: A Core Immune Evasion Strategy of the Virus

Mitochondria serve as both the energy factories of the cell and critical signaling hubs for innate immunity. The mitochondrial antiviral signaling protein (MAVS), anchored on the outer mitochondrial membrane, functions as a central adaptor in the RLR pathway, relaying activation signals from RIG-I/MDA5 to downstream cascades that induce type I interferon production [33]. PEDV has evolved multiple mechanisms to dismantle this signaling platform by inducing mitophagy, thereby systematically suppressing host innate immunity.

The viral non-structural protein Nsp14 is a key effector of this strategy. Yang et al. (2025) [34] demonstrated that Nsp14 simultaneously interacts with the outer mitochondrial membrane protein TOM20 and the autophagy receptor NDP52. In doing so, it acts as a molecular bridge that directly recruits NDP52 to the mitochondrial surface and initiates a selective mitophagy program. Because MAVS is anchored to the outer mitochondrial membrane, it is consequently degraded via the autophagosome–lysosome pathway, effectively blocking RLR signaling and suppressing IFN-β production [35]. Notably, this mechanism bypasses the classical PINK1–Parkin pathway by directly co-opting the substrate recognition function of an autophagy receptor—a strategy that reflects the virus’s sophisticated manipulation of host autophagy. However, these findings currently derive from a single study [34] and await independent corroboration in other cell types and PEDV strains.

PEDV also induces mitophagy through a parallel mechanism involving its N protein and the host E3 ubiquitin ligase TRIM28. Li et al. (2024) [36] found that PEDV infection upregulates TRIM28 expression. TRIM28 then binds to the viral N protein via its RING domain to co-induce mitophagy, which in turn suppresses the JAK–STAT1 signaling pathway and downstream interferon-stimulated genes (ISGs) [37]. Knockdown or knockout of TRIM28 inhibited PEDV replication and restored JAK–STAT1 activity, whereas TRIM28 overexpression had the opposite effect. The mitophagy activator CCCP mimicked TRIM28-mediated STAT1 inhibition, while the inhibitor Mdivi-1 attenuated it. This study not only revealed the key role of the TRIM28–N protein axis in PEDV immune evasion but also proposed a functional chain of “mitophagy–JAK/STAT inhibition–enhanced viral replication”—a model that, while supported by pharmacological and genetic interventions [36], would benefit from confirmation in intestinal epithelial cells and in vivo models. Beyond immunosuppression, PEDV N protein also triggers mitophagy via the classical PINK1/Parkin pathway by ubiquitinating the mitochondrial fusion protein MFN2. This event not only inhibits apoptosis in the early stage of infection but also synergizes with JAK/STAT1 suppression to promote viral replication from multiple angles [38].

Collectively, PEDV constructs a multi-layered mitophagy-based immune evasion network through the coordinated actions of Nsp14, N protein, and the host factor TRIM28. Rather than directly antagonizing individual immune signaling molecules, the virus dismantles the entire mitochondrial signaling platform to achieve a more profound and sustained immunosuppressive effect. The presence of multiple functionally overlapping mitophagy inducers may represent a “redundancy design” that ensures robust execution of this strategy across different infection stages and cell types, shaped by long-term virus–host co-evolution.

### 3.2. Exploiting Autophagic Membranes to Build Replication Platforms: Appropriation of Spatial Resources

A defining feature of the autophagic process is the formation of double-membrane autophagosomes, which provide an ideal physical platform for viral replication [39,40]. For viruses like PEDV that complete RNA replication in the cytoplasm, efficiently enriching and isolating the required viral proteins, host factors, and RNA templates in a relatively confined space is key to enhancing replication efficiency. Autophagic membranes perfectly meet this need—their double-membrane structure both protects viral RNA from degradation by cytoplasmic nucleases and sequesters viral replication products from host pattern recognition receptors [41].

Wang et al. (2022) [42] revealed the signaling pathway by which PEDV actively induces autophagy to appropriate membrane resources. They showed that PEDV infection activates TAK1 kinase, which in turn initiates the TAK1–AMPK/JNK signaling axis. AMPK activation induces phosphorylation of its downstream target ULK1 at serine 777, thereby initiating the autophagic program. This process creates a cellular environment favorable for viral replication, likely by providing membranes for replication complex assembly and by recycling cellular components through autophagic degradation to supply energy and raw materials for viral synthesis [43]. This mechanism reveals how PEDV exploits the host energy-sensing pathway: AMPK is a key kinase for sensing energy deficiency; the virus activates this pathway to “modulate” the cell into a starvation-like state, thereby actively triggering autophagy and providing replication resources for the virus. Studies by Lin et al. (2020) [44] and Zhao et al. (2024) [45] further complemented the molecular mechanisms of PEDV-induced autophagy from another perspective. They found that the viral non-structural protein NSP6 binds to the TLR4 receptor, inhibits the Akt–mTOR signaling pathway, and thereby relieves mTOR-mediated negative regulation of the ULK1 initiation complex, promoting autophagosome formation. Since mTOR is a central hub for cellular nutrient sensing and growth, PEDV pushes the cell into a “catabolic” state by inhibiting this pathway; the massive accumulation of autophagosomes provides ample membrane sources for the assembly of viral replication complexes.

It is worth noting that autophagosome accumulation and autophagic degradation are separable processes. Vargas et al. (2019) [46], in a study on the mechanism of selective autophagy, found that the cargo receptor NDP52, with the assistance of TBK1, can directly recruit the ULK1 complex to ubiquitinated cargo surfaces. This achieves local activation of ULK1 independent of the AMPK/mTOR energy-sensing pathway. This suggests that PEDV-induced autophagy may adopt a dual regulation mode of “signal activation” and “spatial targeting”, providing ULK1 kinase activity via the TAK1–AMPK axis or the PI3K/Akt/mTOR axis (signal activation), while possibly utilizing a mechanism similar to NDP52/TBK1 to precisely target the ULK1 complex at viral replication complexes or damaged organelles (spatial targeting). Such dual regulation ensures that autophagic membrane resources are efficiently and directionally used for viral replication rather than for indiscriminate cellular autophagy. However, whether PEDV directly manipulates the NDP52/TBK1 axis and whether its encoded viral proteins contain receptor-like functional domains remain to be further investigated.

### 3.3. Blocking Autophagic Flux and Inhibiting Selective Autophagy: A Defensive Strategy to Evade Degradation

If inducing autophagosome formation is about “expropriating architectural space” for viral replication, then blocking autophagosome–lysosome fusion and inhibiting autophagic degradation serves to prevent the virus itself from becoming a target of autophagic clearance [47]. PEDV appears to exploit this principle, as genuine degradative autophagy is lethal to the virus, whereas non-degradative autophagic membranes can be exploited. Therefore, the infection tends to lock autophagy in an “intermediate state”—autophagosome accumulation with blocked autophagic flux [48,49].

This model is supported by experimental evidence: PEDV infection leads to accumulation of the autophagy marker LC3-II and elevated p62/SQSTM1 levels, a hallmark of impaired autophagic flux [50]. Blocked flux indicates that autophagosomes fail to fuse with lysosomes, thus sparing viral components from lysosomal degradation. This “bend but don’t break” strategy—initiating autophagy while blocking its completion—is a balancing act employed by multiple viruses [51].

Beyond passively blocking flux, PEDV also actively dismantles the host’s selective autophagic defense. Host cells can recognize the coronavirus envelope (E) protein via selective autophagy receptors such as NBR1 and CCDC50, delivering it to the autophagosome–lysosome pathway for degradation. To counteract this, the non-structural protein NSP5 of porcine deltacoronavirus (PDCoV) cleaves NBR1 [52] or CCDC50 [53], disrupting their receptor function. Notably, NSP5 from multiple coronaviruses—including PEDV and SARS-CoV-2—possesses this cleavage activity, suggesting a conserved immune evasion strategy within the coronavirus family. However, the evidence for NSP5-mediated cleavage in the context of PEDV infection remains indirect, as most data derive from PDCoV studies [52,53]; direct demonstration in PEDV-infected cells is needed to confirm that this strategy is broadly utilized.

A recent study by Li et al. (2026) [54] revealed an additional layer of sophistication: PEDV nsp14, a protein with S-adenosyl-L-methionine (SAM)-binding capability, upregulates autophagy-related genes (ATG4A, ATG5, ATG7) and stabilizes ATG4A mRNA, promoting GABARAP lipidation and mitophagy, while simultaneously suppressing LC3B-mediated antiviral autophagy (virophagy) by degrading SQSTM1/p62. This shift from “LC3B-mediated defensive autophagy” to “GABARAP-mediated pro-viral mitophagy” represents a remodeling of the host autophagic system—favoring a branch beneficial to the virus while suppressing a restrictive one. This “reprogramming” model is conceptually important but currently rests on a single study [54]; its generalizability to other PEDV strains and cell types remains to be established.

Collectively, these findings support a model in which PEDV actively blocks autophagic flux and subverts selective autophagy to evade host defense. Nevertheless, several caveats merit consideration. First, evidence for flux blockade relies heavily on pharmacological inhibitors (bafilomycin A1, chloroquine), which may have off-target effects on endosomal trafficking and immune signaling. Second, the extent of flux blockade appears to vary across cell types (Vero vs. IPEC-J2) and PEDV strains, suggesting that viral genetic heterogeneity may influence its efficiency. Third, it remains unclear whether flux blockade is an actively encoded viral function or a secondary consequence of ER stress-induced autophagy dysregulation; distinguishing between these possibilities will require systematic comparison of isogenic viral strains differing only in candidate genes (e.g., nsp5, nsp6, or ORF3). Finally, while autophagosome accumulation is consistently observed, its functional contribution to viral replication has largely been inferred from pharmacological inhibition of autophagy initiation; direct visualization of viral replication complexes on autophagic membranes in living cells would provide more definitive evidence. Despite these limitations, the convergent evidence from multiple independent studies strongly supports the conclusion that PEDV utilizes a triple-pronged strategy—inducing autophagosome formation (resource expropriation), blocking autophagic flux (evading clearance), and reprogramming autophagic branches (optimized utilization)—to convert the host autophagic system into an efficient replication platform. This multi-layered strategy reflects the virus’s refined manipulation of host autophagy, shaped by long-term evolutionary pressures.

## 4. Molecular Mechanisms by Which Autophagy Suppresses PEDV Replication: A Multi-Level Host Defense System

Facing PEDV’s manipulation of the autophagic system, host cells are not passive victims but have developed a sophisticated defense system that actively recognizes, tags, and degrades viral components via autophagic mechanisms. Although the host factors involved are numerous and diverse, their mechanisms of action share a highly unified molecular logic: the “ubiquitination–autophagy receptor–degradation” axis [55,56].

### 4.1. The “Ubiquitination—Autophagy Receptor—Degradation” Axis: Core Logic of Host Defense

**Figure 3:**
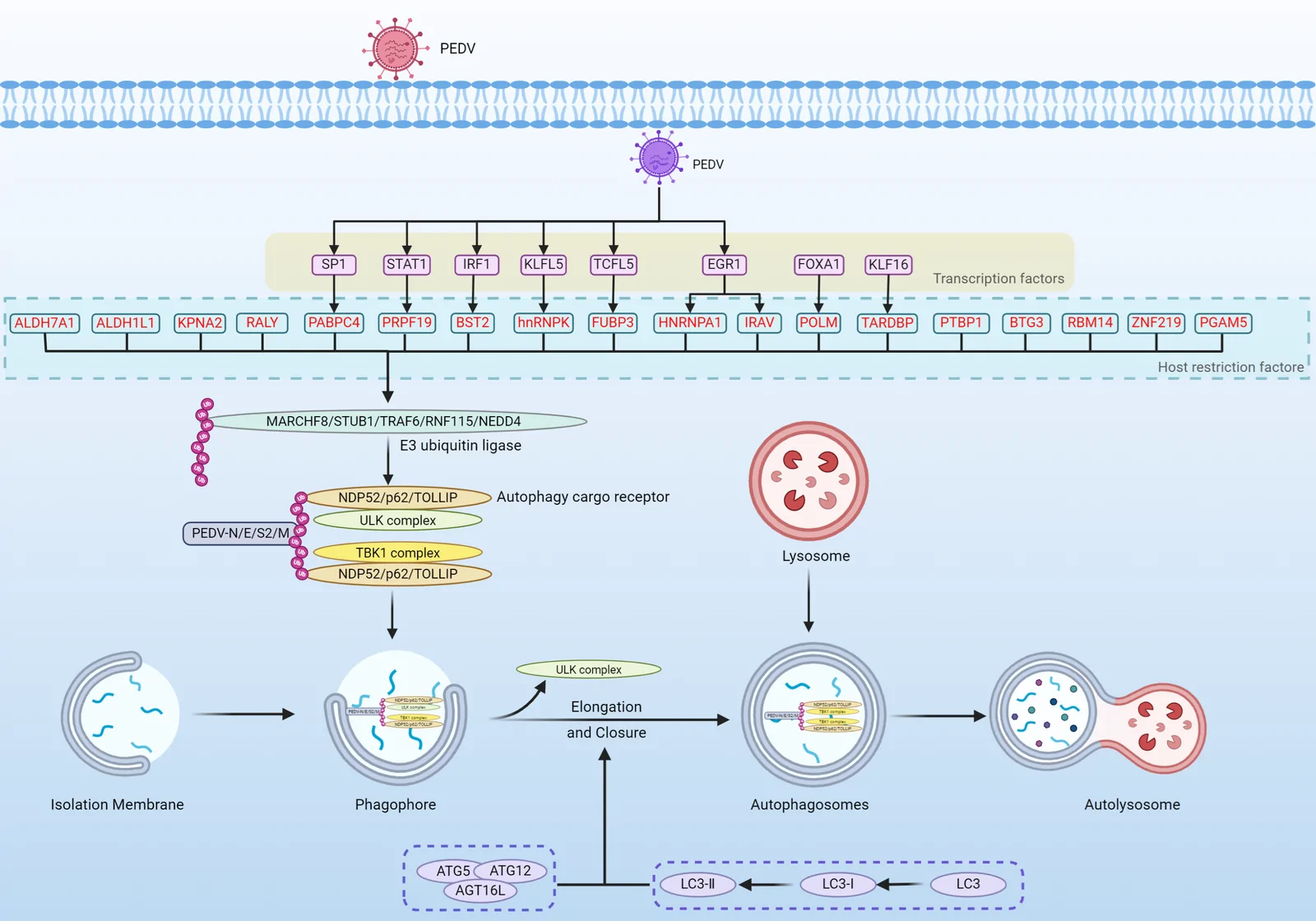
Schematic illustration of host restriction factor-mediated selective autophagic degradation of PEDV proteins. PEDV structural proteins—primarily the nucleocapsid (N) protein, but also S2, E, and M—are recognized by host restriction factors (e.g., ALDH1L1, RBM14, BTG3, PGAM5, and RNF115). These factors recruit E3 ubiquitin ligases (including MARCHF8, STUB1, TRAF6, and RNF115) to catalyze K63-linked polyubiquitination of viral proteins. The ubiquitinated cargo is then recognized by autophagy receptors—predominantly NDP52, p62, and TOLLIP—which simultaneously bind the cargo via their ubiquitin-associated (UBA) domain and interact with autophagosomal membrane proteins LC3/GABARAP via their LC3-interacting region (LIR) motif. This dual interaction delivers the cargo to the nascent autophagosome. The autophagosome subsequently fuses with the lysosome, forming an autolysosome where the enclosed viral proteins are degraded by acidic hydrolases, thereby restricting PEDV replication. Additionally, several restriction factors (e.g., hnRNP K, FUBP3, TARDBP, PTBP1) also activate type I interferon signaling, providing a parallel antiviral mechanism (not depicted). For detailed mechanisms of individual restriction factors, see Table 1 and Section 3.

Over the past five years, more than 16 host restriction factors have been identified that inhibit PEDV replication through selective autophagy, including ALDH1L1 [57], POLM [58], BTG3 [59], RBM14 [60], ZNF219 [61], IRAV [62], hnRNP K [63], FUBP3 [64], TARDBP [65], BST2 [66], PRPF19 [67], PTBP1 [68], PABPC4 [69], RALY [70], KPNA2 [71], and PGAM5 [72] (Table 1). Despite their structural and functional diversity, these factors operate through a shared three-step molecular logic: (i) Substrate recognition and ubiquitination labeling. Host restriction factors ubiquitinate PEDV structural proteins—primarily the N protein, but also S, E, and M—as well as non-structural proteins such as NSP8, either by direct binding or by recruiting E3 ubiquitin ligases. Among these, membrane associated ring-CH-type finger 8 (MARCHF8) is the most frequently mobilized E3 ligase, participating in the mechanisms of at least eight restriction factors, underscoring its role as a core effector in the host’s autophagic defense network. Additional E3 ligases—including STUB1, TRAF6, RNF115 [73], and NEDD4 [74]—are engaged by specific restriction factors, reflecting the diversity and redundancy of the host defense system. (ii) Recognition by autophagy receptors and substrate delivery. Ubiquitinated viral proteins are recognized by specific autophagy receptors, forming cargo–receptor complexes. NDP52 and p62 serve as the principal autophagy receptors, responsible for distinguishing different ubiquitin chain types or substrate features [75,76]. Tollip functions as another important receptor, playing a key adaptor role in RNF115-mediated N protein degradation [73]. Autophagy receptors recognize ubiquitinated substrates via their C-terminal UBA domain and simultaneously interact with the autophagosomal membrane proteins (LC3/GABARAP) through their N-terminal LIR motif, enabling precise cargo delivery to autophagosomes. (iii) Autophagosome–lysosome degradation. Viral proteins enclosed in autophagosomes are ultimately delivered to lysosomes and degraded by acidic hydrolases, thereby blocking the viral life cycle (Figure 3). Notably, several restriction factors (e.g., EGR1–IRAV, TARDBP, PTBP1, FUBP3, hnRNP K) also upregulate type I interferon signaling in addition to activating autophagic degradation, synergistically inhibiting viral replication through dual mechanisms.

While the convergence of multiple factors on MARCHF8-mediated degradation and the broad-spectrum anti-coronavirus activity of PABPC4 [69] lend confidence to the overall framework, it should be noted that several of the restriction factors listed in Table 1—particularly RNF115 [73] and NEDD4 [74]—have been characterized in single studies to date. Independent validation in additional cell types and experimental systems will be essential to confirm their general significance.

This “ubiquitination–autophagy receptor–degradation” axis reveals the core logic of the host’s antiviral defense: by combinatorially employing different E3 ligases, autophagy receptors, and effector proteins, the host can flexibly counter diverse viral immune evasion strategies. Even if the virus evolves to inhibit one E3 ligase or receptor, alternative factors can maintain defensive function.

### 4.2. Synergistic Defense Network of Host Restriction Factors

Importantly, the above restriction factors do not act in isolation but form a cooperative, mutually reinforcing defense network. This synergy is reflected at multiple levels: (i) Complementarity of target coverage. Different restriction factors target different viral proteins or different regions of the same protein. For example, ALDH1L1 can degrade both N and E proteins [57], POLM targets three structural proteins N, S2, and M [58], while BTG3 mainly targets the S2 protein [59]. This target diversity ensures comprehensive suppression of multiple viral structural components. Notably, the N protein is the most intensively studied target, with at least 12 restriction factors shown to promote its degradation via the autophagic pathway, forming a “saturating defense” against this key viral protein. (ii) Cross-enhancement of signaling pathways. Several restriction factors (e.g., hnRNP K, FUBP3, TARDBP, PTBP1) not only activate autophagic degradation but also promote type I interferon production via the MyD88–TRAF3/6–IRF3 axis [63,64,65,68]. Upregulation of interferon signaling not only directly inhibits viral replication but also further induces the expression of additional antiviral factors such as BST2 and PRPF19, forming a positive feedback loop that amplifies the antiviral effect. (iii) Implications of broad-spectrum anti-coronavirus activity. Jiao et al. (2021) [69] found that PABPC4 can degrade the N proteins of multiple coronaviruses (including MHV (β-coronavirus), IBV (γ-coronavirus), and PDCoV) through selective autophagy, demonstrating broad-spectrum anti-coronavirus activity. This finding has important implications: although different coronaviruses have their own characteristics in immune evasion, their structural proteins (especially the N protein) may share common “vulnerabilities” when facing the host autophagic system. Deep understanding of this common mechanism may provide a theoretical basis for developing broad-spectrum anti-coronavirus drugs.

It is worth noting that the functions of host restriction factors are dynamically regulated by viral countermeasures. For example, PGAM5 protein levels decrease while its mRNA levels increase after PEDV infection, suggesting that the virus may promote the degradation of this protein through some mechanism [72]. This dynamic “host defense–viral counter defense” interplay determines the actual strength of autophagy mediated antiviral effects during infection.

## 5. The Endoplasmic Reticulum Stress–Autophagy Axis: The Central Hub of the Interplay

The previous two sections have described the pro-viral and anti-viral functions of autophagy during PEDV infection, but an important question remains: how are these two seemingly opposing functional axes coordinated, and where do they converge? Growing evidence indicates that the endoplasmic reticulum (ER) stress–autophagy axis serves precisely as the central hub for this interplay.

### 5.1. Molecular Mechanisms of PEDV-Induced ER Stress

The endoplasmic reticulum (ER) is the primary site for the synthesis, folding, and maturation of viral glycoproteins. During coronavirus replication, massive production of structural proteins—particularly the heavily glycosylated S protein—readily overwhelms the ER folding capacity, leading to accumulation of unfolded/misfolded proteins and triggering ER stress, which activates the unfolded protein response (UPR) [77]. PEDV infection induces ER stress in host cells, as evidenced by upregulation of GRP78/BiP and activation of all three UPR signaling branches: PERK, IRE1, and ATF6 [78,79]. Multiple viral proteins contribute to this process: ORF3 acts through the PERK–eIF2α pathway [79], while the N, E, and Nsp6 proteins have also been implicated [78,80,81]. This functional redundancy may ensure effective ER stress induction across different infection stages and cell types.

Recent work has elucidated an upstream trigger of PEDV-induced ER stress. Mo et al. (2026) [82] demonstrated that the viral proteins NSP1 and NSP2 cooperatively enhance KEAP1–NRF2 binding, shifting NRF2 ubiquitination from K63-linked (stabilizing) to K48-linked (degradative), thereby promoting proteasomal degradation of NRF2. As the master transcriptional regulator of antioxidant responses, NRF2 loss impairs glutathione synthesis and suppresses the NRF2/HO-1 antioxidant pathway, leading to substantial ROS accumulation [83], which in turn induces ER stress and downstream autophagy. Notably, this NSP1/NSP2–NRF2–ROS axis currently derives from a single study [82] and awaits independent validation in additional cell models and PEDV strains.

Collectively, these discrete findings—NRF2 degradation by NSP1/NSP2 [82], ROS-driven ER stress [78,81,83], and ER stress-induced autophagy [77,78]—can be integrated into a coherent signaling cascade: viral protein → NRF2 degradation → ROS accumulation → ER stress → autophagy. While each step has been experimentally validated in separate studies, the complete cascade as a unified pathway awaits direct experimental confirmation within a single experimental system.

### 5.2. Fine Regulation of the ER Stress–Autophagy Axis by the Host

Facing PEDV-induced activation of the ER stress–autophagy axis, host cells are not passive but finely regulate signal intensity and direction through multiple regulatory factors. Xu et al. (2022) [84] found that the host protein DDX6, a negative regulator of autophagy, is downregulated during PEDV infection, thereby relieving its inhibition of autophagy. DDX6 suppresses autophagy by promoting the degradation of ATG-related mRNAs and maintaining mTOR phosphorylation levels, whereas PEDV-induced ER stress reduces DDX6 protein levels. This mechanism reveals the ability of host cells to maintain dynamic balance between ER stress and autophagy—timely downregulation of DDX6 provides flexibility for moderate autophagy activation under stress conditions. Li et al. (2025) [85] found that angiotensin-converting enzyme 2 (ACE2) can inhibit PEDV-induced ER stress and autophagy by reducing intracellular ROS production, thereby alleviating cell damage. This finding suggests that ACE2 not only serves as a receptor for PEDV entry but may also act as a negative regulator of the ER stress–autophagy axis at the post-infection stage, playing a protective role. However, the non-linear relationship between ACE2 expression level and the outcome of PEDV infection—overly high expression may favor viral entry, while too-low expression may lead to excessive ER stress activation and cell damage—warrants further investigation.

### 5.3. The “Double Edged Sword” Effect of the ER Stress–Autophagy Axis

The ER stress–autophagy axis exerts a classic “double-edged sword” effect during PEDV infection. On one hand, moderate autophagy activation benefits viral replication; on the other, excessive or sustained autophagy may shift toward apoptosis or enhanced antiviral immunity. The pro-viral role is supported by direct experimental evidence. Sun et al. (2021) [78] demonstrated that PEDV-induced autophagy via ROS-activated PERK and IRE1 pathways promotes viral replication, likely by providing autophagic membranes for replication complex assembly, supplying raw materials from degradation products, and contributing to immunosuppression. Zou et al. (2019) [79] further established a direct functional link between ER stress and autophagy induction, showing that ORF3 protein-induced ER stress—dependent on the PERK–eIF2α pathway—promotes LC3-I to LC3-II conversion. Conversely, sustained ER stress–autophagy activation may exceed the virus’s regulatory capacity, leading to enhanced cell damage, inflammatory cytokine release, or selective autophagic degradation of viral proteins. ER stress itself may also promote MHC class I-mediated antigen presentation via the IRE1–XBP1 pathway, enhancing adaptive immunity. Importantly, while the pro-viral role is experimentally validated, the proposed “functional switch” from pro-viral to anti-viral autophagy—and the threshold conditions governing it—remains inferential. This inference is grounded in the well-established principle that excessive autophagy can exceed viral regulatory capacity, but direct experimental validation in the PEDV context is currently lacking. We therefore present this duality as a testable hypothesis rather than a definitive conclusion and highlight it as a priority for future investigation (see Section 6). The ultimate outcome of infection likely depends on the activation intensity, duration, and signal branch selection of the ER stress–autophagy axis, with the virus favoring moderate activation and the host attempting to tip the balance in the anti-viral direction.

## 6. Targeting Autophagy for Anti-PEDV Strategies: From Mechanism to Application

**Figure 4:**
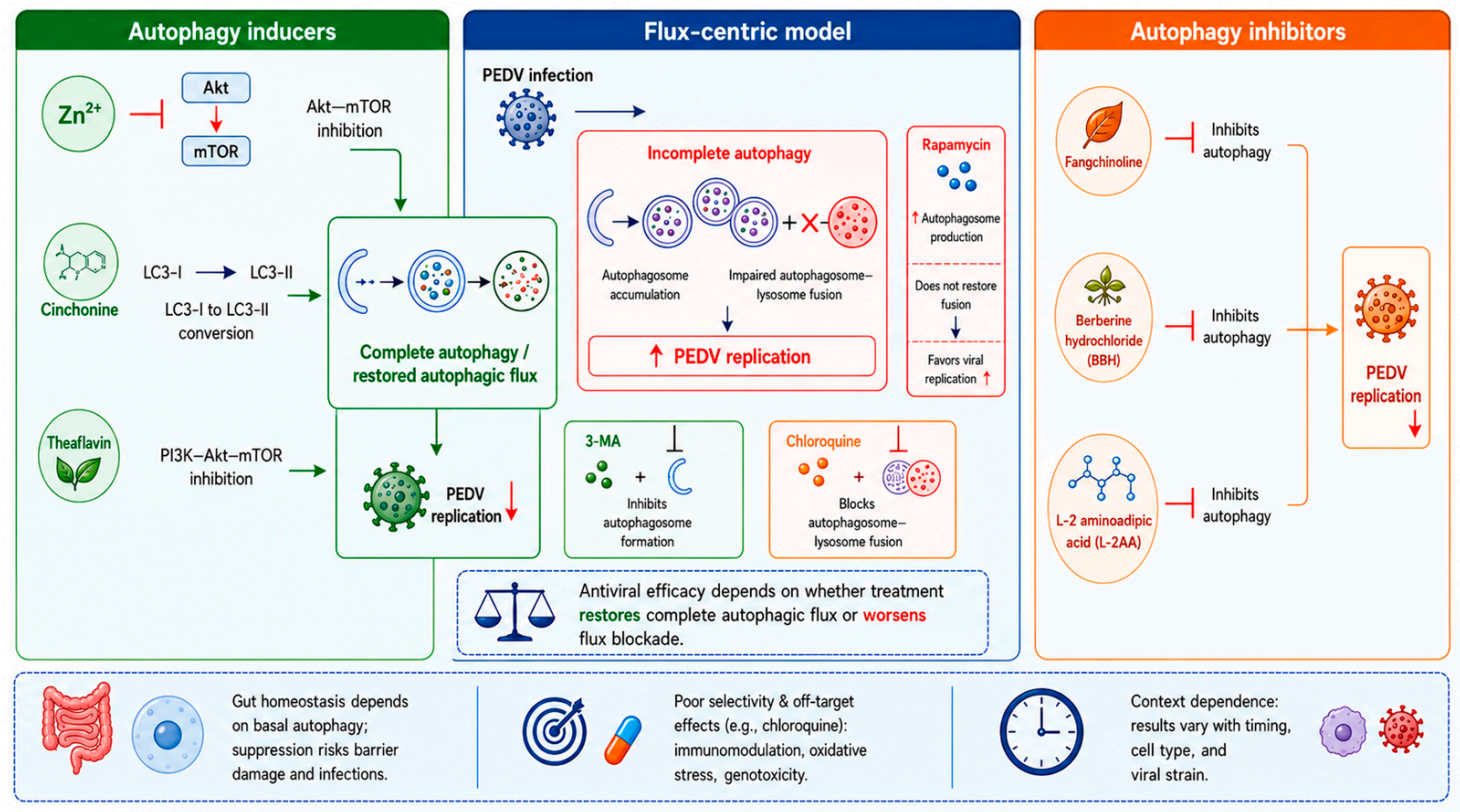
Autophagy-targeting strategies for the inhibition of porcine epidemic diarrhea virus (PEDV) replication. Autophagy-modulating compounds can exert anti-PEDV effects through distinct mechanisms. On the left, autophagy inducers, including Zn^2+^, cinchonine, and theaflavin, promote autophagy through modulation of the Akt/mTOR or PI3K/Akt/mTOR pathways or by enhancing LC3-I-to-LC3-II conversion. When these interventions facilitate complete autophagic flux and autophagosome–lysosome degradation, PEDV replication is suppressed. In the central flux-centric model, PEDV infection induces incomplete autophagy characterized by autophagosome accumulation and impaired autophagosome–lysosome fusion, thereby favoring viral replication. Rapamycin further increases autophagosome formation without restoring lysosomal fusion and may consequently enhance viral replication, whereas 3-methyladenine (3-MA) inhibits autophagosome formation and chloroquine blocks autophagosome–lysosome fusion. These observations highlight that the antiviral consequence of autophagy modulation depends on whether autophagic flux is restored or further disrupted. On the right, autophagy inhibitors, including fangchinoline, berberine hydrochloride (BBH), and L-2-aminoadipic acid (L-2AA), suppress PEDV replication by interfering with autophagy exploited by the virus. However, therapeutic manipulation of autophagy remains constrained by the requirement for basal autophagy in intestinal homeostasis, the limited selectivity and off-target effects of pharmacological modulators, and context-dependent responses related to treatment timing, cell type, and viral strain.

Based on an in-depth understanding of the molecular mechanisms underlying the interplay between autophagy and PEDV, researchers have begun to explore the possibility of inhibiting viral replication by pharmacologically manipulating the autophagic pathway. A variety of compounds with anti-PEDV activity have been identified, and their mechanisms of action are closely related to the regulation of autophagy, though the direction and targets of regulation vary, reflecting the multidimensional role of autophagy during PEDV infection (Table 2 and Figure 4).

### 6.1. Antiviral Application of Autophagy Inducers

Some compounds exert anti-PEDV effects by inducing or enhancing autophagy. Zn^2+^ is a representative example. Wang et al. (2025) [86] found that Zn^2+^ induces autophagy by inhibiting the Akt–mTOR signaling pathway, thereby suppressing PEDV replication. Mechanistically, Zn^2+^ antagonizes PEDV induced Akt phosphorylation and mTOR activation, relieving mTOR mediated negative regulation of autophagy and consequently promoting autophagic flux. This finding has significant translational potential, as zinc preparations are common feed additives that combine nutritional supplementation with antiviral functions. Cinchonine at non cytotoxic concentrations significantly inhibits PEDV infection, mainly acting at the early stage of viral replication. Cinchonine induces cellular autophagy (increased conversion of LC3-I to LC3-II), and the autophagy inhibitor 3-MA markedly attenuates its antiviral effect [87]. Theaflavin suppresses PEDV replication by inhibiting the PI3K/Akt/mTOR signaling pathway, dose-dependently inhibiting viral internalization and replication [91]. Given that this pathway is a key negative regulator of autophagy, the antiviral effect of theaflavin is likely closely related to autophagy induction.

The above examples raise a seemingly paradoxical question: why do compounds that induce autophagy inhibit PEDV replication, while PEDV itself can also benefit from autophagy induction? We propose that this paradox can be resolved by examining the integrity of autophagic flux rather than the mere induction of autophagy. PEDV infection establishes a state of “incomplete autophagy” characterized by autophagosome accumulation but impaired autophagosome–lysosome fusion (Section 3.3). Under this condition, rapamycin further amplifies autophagosome production without restoring fusion, thereby providing additional membranous platforms for viral replication. In contrast, compounds such as Zn^2+^ and cinchonine appear to induce “complete autophagy” with unimpaired flux, enabling lysosomal degradation of viral components. This interpretation is consistent with the observation that inhibitors blocking autophagosome formation (e.g., 3-MA) reduce viral replication, whereas inhibitors blocking autophagosome–lysosome fusion (e.g., chloroquine) have the opposite effect [50,89]. We therefore propose that the antiviral efficacy of autophagy modulating agents depends not on whether they induce or inhibit autophagy per se, but rather on whether they shift the flux toward completion (antiviral) or exacerbate the blockade (proviral). This flux-centric model provides a testable framework for future drug screening: candidate compounds should be evaluated not only for their effects on LC3-II levels but also for their ability to restore autophagosome–lysosome fusion in PEDV-infected cells.

### 6.2. Antiviral Application of Autophagy Inhibitors

In contrast to autophagy inducers, several compounds exert anti-PEDV effects by inhibiting autophagy. Fangchinoline dose-dependently suppresses PEDV infection at non-cytotoxic concentrations (EC_50_ = 0.67 μM) by regulating autophagy-related proteins and blocking autophagic flux [88]. Berberine hydrochloride (BBH) exhibits anti-PEDV activity both in vitro and in vivo, dose-dependently reducing LC3-II expression, and the autophagy activator rapamycin partially reverses its antiviral effect, indicating that autophagy inhibition contributes to its mechanism [89]. Similarly, L-2-aminoadipic acid (L-2AA) suppresses PEDV replication through autophagy inhibition, an effect reversible by rapamycin [90].

The rationale for autophagy inhibition as an antiviral strategy is straightforward: by preventing autophagosome formation or blocking autophagic flux, the virus is deprived of the membranous platforms and immune evasion mechanisms it exploits. However, translating this strategy into safe and effective clinical or field applications faces substantial challenges. First, the intestinal epithelium—the primary target of PEDV—is exquisitely dependent on basal autophagy for homeostasis. Genetic ablation of core autophagy genes such as *Atg16L1* or *Atg5* in intestinal epithelial cells disrupts Paneth cell granule morphology and antimicrobial peptide secretion [92,93,94,95], compromises protection against enteric pathogens [96,97], and impairs intestinal stem cell regeneration following injury [98,99]. Systemic or prolonged autophagy suppression could therefore undermine gut barrier integrity and increase susceptibility to secondary infections.

Second, the pharmacological tools commonly used to inhibit autophagy are far from selective. Chloroquine (CQ), for instance, blocks autophagosome–lysosome fusion but also exerts anti-inflammatory and immunomodulatory activities [100,101], stimulates iNOS expression and NO synthesis [102,103], and induces oxidative stress upon prolonged use [104]. These off-target effects can independently influence viral replication or host cell responses, confounding the interpretation of studies that attribute antiviral activity solely to autophagy inhibition. Some concentrations of CQ have also been associated with genotoxicity in vivo [105,106], raising additional safety concerns for long-term use.

Third, the outcome of autophagy modulation is highly context-dependent: the same intervention may produce opposing effects depending on the timing of administration, the cell type, and the viral strain [50,89]. This variability complicates the design of standardized therapeutic protocols and underscores the need for a nuanced understanding of autophagy dynamics during PEDV infection.

### 6.3. Strategy Comparison and Future Directions

The observation that both autophagy inducers (Section 5.1) and inhibitors can suppress PEDV replication may initially seem paradoxical, yet it reflects the stage-specific and bidirectional nature of autophagy functions during infection. Rather than pursuing a simplistic “activate or inhibit” approach, future strategies should aim for precision regulation of autophagy—tailoring interventions to the specific status of autophagic flux, the identity of the cargo being degraded, and the stage of infection.

Several promising directions merit attention. First, the development of selective modulators that distinguish autophagosome formation from autophagic degradation could enable more nuanced control: for example, agents that specifically counteract virus-induced blockade of autophagosome–lysosome fusion would restore degradative function without further amplifying autophagosome production [107]. Second, targeting virus–host interaction interfaces—such as the Nsp14–NDP52 complex, the N protein–TRIM28 axis, or NSP5-mediated cleavage of autophagy receptors—could disrupt viral subversion of autophagy while leaving basal cellular functions intact [108]. Third, combination strategies that pair autophagy modulators with interferons, neutralizing antibodies, or other antiviral agents may achieve synergistic efficacy through multi-target engagement. Finally, tissue-specific delivery—for instance, oral formulations with limited systemic absorption—could help restrict drug activity to the intestinal epithelium, thereby minimizing off-target effects on systemic autophagy while preserving antiviral activity at the primary site of PEDV replication.

Addressing these challenges and pursuing these directions will require not only more selective chemical tools but also robust biomarkers for monitoring autophagic flux in vivo, enabling the rational design of autophagy-targeted interventions that are both effective and safe.

## 7. Conclusions and Outlook

The interplay between autophagy and PEDV epitomizes the long-term co-evolution of virus and host. Based on a systematic review of current research, this article reveals the intrinsic logic of this interplay: PEDV utilizes a triple-pronged strategy—subverting mitophagy to suppress innate immunity, exploiting autophagic membranes to build replication platforms, and blocking autophagic flux to evade degradation—to convert the host autophagic system into an efficient pro-viral tool. In response, host cells mobilize a diverse array of restriction factors along the unified molecular axis of “ubiquitination–autophagy receptor–degradation” to launch multi-level autophagy-dependent clearance of viral proteins. These two functional axes converge at the oxidative stress–ER stress–autophagy hub, where viral NSP1/NSP2 trigger the cascade by inhibiting NRF2, while host factors such as DDX6 and ACE2 finely regulate it. Importantly, this integrated framework offers a unifying explanation for the seemingly contradictory reports on whether autophagy promotes or restricts PEDV infection: the outcome depends on whether an intervention exacerbates the virus-induced blockade of autophagosome–lysosome fusion (pro-viral) or restores flux completion (anti-viral). This flux-centric model thus provides a coherent lens for interpreting past findings and designing future experiments.

A caveat that applies broadly to this field is that many of the mechanistic models discussed herein—particularly those involving recently identified host factors (e.g., RNF115, NEDD4, PGAM5) and viral proteins (e.g., Nsp14, NSP1/NSP2)—are derived from single studies that have not yet been independently replicated. While the convergence of multiple lines of evidence on common themes (e.g., K63-linked ubiquitination, NDP52-mediated cargo recognition, and MARCHF8-dependent degradation) lends credence to the overall framework, caution is warranted when interpreting findings that currently lack validation across different experimental systems, cell types, or viral strains. Encouraging independent replication of these key findings should be a priority for the field.

In addition, several limitations of this review itself should be acknowledged. First, the vast majority of mechanistic studies reviewed herein are derived from in vitro cell culture models, and their physiological relevance requires confirmation in vivo. Second, while several compounds have shown promising anti-PEDV activity in vitro, only a handful have been evaluated in vivo, and none have progressed to clinical or field applications; the pharmacokinetic, safety, and efficacy profiles of autophagy-modulating agents in pigs remain largely unexplored. Third, this review focuses on the mechanistic interplay between autophagy and PEDV replication and does not directly address vaccine development strategies, although understanding how PEDV manipulates host autophagy could inform the rational design of attenuated vaccines.

Despite these advances, several core questions remain to be explored in depth: (i) Precise mechanisms of autophagic flux regulation. How does PEDV lock autophagy in an “intermediate state”—activating autophagosome formation while blocking its degradative function—and what are the molecular switches? Several coronaviruses block autophagosome–lysosome fusion via SNARE interactions or lysosomal acidification interference [109]; whether PEDV utilizes similar or distinct strategies requires systematic investigation. (ii) Molecular code for selective autophagy substrate recognition. How do host restriction factors specifically recognize viral proteins among numerous cellular proteins? How do the type (K48 vs. K63), site, and topology of ubiquitination influence autophagy receptor choice [110]? Do structural features of viral proteins—such as intrinsically disordered regions or phase separation propensity—determine their susceptibility to autophagic degradation [111]? Addressing these questions will require integrating structural biology, ubiquitination-modified proteomics, and in vitro reconstitution approaches. (iii) Regulatory network of the ER stress–autophagy axis. How does this axis achieve the functional switch from pro-viral to anti-viral during infection, and what are the threshold conditions? Do the three UPR pathways (IRE1, PERK, ATF6) play distinct or synergistic roles? The IRE1 pathway regulates autophagy-related gene expression via XBP1 and autophagy initiation via JNK; its net effect likely depends on infection stage and cellular context, necessitating time-resolved systems that simultaneously monitor UPR activity, autophagic flux, and viral replication. (iv) Genotype specificity of autophagy–PEDV interactions. PEDV strains are classified into G1 (classical strains) and G2 (pandemic strains), with G2 having diversified into G2a, G2b, and G2c sub-lineages, and recent epidemiological surveys indicating a marked increase in G2c-associated outbreaks [16,112]. These genogroups differ in genomic sequences—particularly in the hypervariable S gene—and in pathogenicity, with G2 strains generally exhibiting higher virulence [23,50]. Current evidence, though limited, suggests genotype-dependent differences may exist: transcriptomic analyses revealed that the virulent GDS01 strain (G2) enriched autophagy- and apoptosis-associated genes, whereas the avirulent HX strain predominantly enriched immunity- and inflammation-related genes [113]. Similarly, autophagy facilitates replication of the virulent CH/YNKM-8/2013 strain (G2) at late infection stages, while the CV777 vaccine strain (G1) engages autophagy with potentially different kinetics [32,112]. The PEDV strain JS-2013 has been reported to inhibit autophagy by activating the PI3K–AKT pathway, in contrast to other strains that induce autophagy to promote replication [77]. However, systematic comparative analyses across multiple G1 and G2 strains remain scarce, and the inconsistent findings in the literature may be partially attributable to strain-specific differences [50]. Future studies should systematically compare representative strains from both genogroups under standardized conditions to determine whether autophagy manipulation mechanisms are conserved or strain-specific. (v) Translational perspectives: bridging the in vitro–in vivo gap. The majority of mechanistic findings discussed in this review are derived from cell culture models, primarily Vero, LLC-PK1, and IPEC-J2 cells [29]. While these systems have been invaluable for dissecting molecular pathways, their translation to in vivo settings remains challenging. Nevertheless, key in vitro findings have been successfully translated to the physiological context: NEDD4 is upregulated in PEDV-infected piglet intestinal tissues, confirming its antiviral role in vivo [74]; the small-molecule compound Cyclo-C significantly reduces mortality, lowers viral loads, and protects intestinal villus architecture in PEDV-challenged neonatal piglets [114]; and rapamycin has been suggested as a potential prophylactic strategy [115]. However, several obstacles complicate extrapolation: the intestinal microenvironment—including gut microbiota, mucus layer, and immune cell populations—cannot be fully recapitulated in monolayer cultures [99,116]; pharmacokinetics and bioavailability differ substantially in vivo [117]; and genotype-dependent strain differences may affect generalizability [50]. To bridge this gap, a tiered approach is recommended: validation in porcine intestinal organoids, which preserve epithelial architecture and differentiation [118,119]; ex vivo studies using intestinal explant cultures to assess tissue-level responses [120]; and controlled in vivo challenge studies in piglets with comprehensive endpoints, including viral load quantification, histological assessment, and monitoring of autophagic flux. The development of selective autophagy modulators with minimal off-target effects, robust biomarkers for monitoring autophagic flux in vivo, and optimized delivery strategies (e.g., oral formulations with limited systemic absorption) are critical steps toward field-applicable antiviral strategies.

From a broader perspective, research on PEDV–autophagy interplay has implications beyond this single pathogen. Notable conservation exists in autophagy-manipulation strategies across the coronavirus family—mechanisms such as NSP5-mediated cleavage of autophagy receptors, nsp14-induced mitophagy, and N protein-mediated inhibition of autophagic degradation have all been reported in SARS-CoV-2 and MHV. In-depth analysis of PEDV as a model system may thus reveal common principles of coronavirus–host interactions and inform the development of broad-spectrum anti-coronavirus strategies.

## Figures and Tables

**Table 1:** Host restriction factors mediating selective autophagic degradation of PEDV proteins.

Host Restriction Factor	Targeted Viral Protein	Recruited E3 Ubiquitin Ligase	Cargo Receptor	Concurrent IFN Pathway Activation	Reference
ALDH1L1	N, E	STUB1	TOLLIP	Not reported	[57]
POLM	N, S2, M	MARCHF8	p62	Not reported	[58]
BTG3	S2	MARCHF8	NDP52	Not reported	[59]
RBM14	N	Not specified	p62	Yes (MAVS pathway)	[60]
ZNF219	S2, M, N	TRAF6	p62	Not reported	[61]
IRAV	N	MARCHF8	Not specified	Not reported	[62]
hnRNP K	N	MARCHF8	NDP52	Yes (MyD88 pathway)	[63]
FUBP3	N	MARCHF8	NDP52	Yes (TRAF3 pathway)	[64]
TARDBP	N	MARCHF8	NDP52	Yes (MyD88-TRAF3 pathway)	[65]
BST2	N	MARCHF8	NDP52	No	[66]
PRPF19	N	MARCHF8	NDP52	Yes (STAT1 regulation)	[67]
PTBP1	N	MARCHF8	NDP52	Yes (MyD88 pathway)	[68]
PABPC4	N (broad-spectrum against coronaviruses)	MARCHF8	NDP52	Not reported	[69]
RALY	N	MARCHF8	NDP52	No	[70]
KPNA2	E	Not specified	Not specified	Not reported	[71]
PGAM5	N	STUB1	p62	No	[72]

**Table 2:** Active substances targeting autophagy with anti-PEDV effects and their functional characteristics.

Substance	Autophagy Modulation	Molecular Targets/Signaling Pathways	Effects on Autophagic Flux	Effective Concentration	Functional Characteristics (Cell Models/Major PEDV Inhibitory Stages)	Reference
Zinc ion (Zn2+)	Induce autophagy	Akt-mTOR pathway; VPS34/Beclin1 complex	Enhance autophagic flux; upregulate LC3-II and downregulate p62	6.4~160 μM	Vero-E6, PK-15 cells; mainly inhibit PEDV attachment, internalization and replication	[86]
Cinchonine	Induce autophagy	Undefined upstream autophagy pathway; independent of endoplasmic reticulum stress	Enhance autophagic flux; accelerate the conversion of LC3-I to LC3-II	20~100 μM	Vero CCL81, LLC-Vero CCL81, LLC-PK1, ST cells; predominantly act on the early stage of PEDV infection	[87]
Fangchinoline	Inhibit autophagy	Autophagosome–lysosome fusion process	Block autophagic flux; induce accumulation of both LC3-II and p62	2.5~20 μM	IPEC-J2 cells; inhibit PEDV attachment, internalization and replication; no obvious effect on viral release	[88]
Berberine hydrochloride	Inhibit autophagy	Undefined upstream pathway	Suppress autophagosome formation and reduce LC3-II expression	10~120 μM	Vero-E6 cells and suckling piglets; inhibit the entire PEDV life cycle, with the most potent efficacy against viral attachment and internalization	[89]
L-2-aminoadipic acid (L-2AA)	Inhibit autophagy	ALDH7A (key enzyme of lysine metabolism)	Suppress complete autophagic flux; decrease LC3-II and increase p62 levels	20~100 μM	LLC-PK1, Vero cells; mainly block PEDV release and slightly inhibit viral attachment	[90]
Theaflavin (TF)	Indirectly regulate autophagy via pathway inhibition	PI3K/Akt/mTOR pathway; directly bind to PEDV N protein and Nsp5	Modulate cellular autophagy by suppressing PI3K/Akt/mTOR pathway activity	25~100 μM	Vero, IPI-HB1 cells; mainly inhibit PEDV internalization and replication	[91]

## Data Availability

No new data were created or analyzed in this study. Data sharing is not applicable to this article.

## Abbreviations

The following abbreviations are used in this manuscript:

3-MA	3-methyladenine
ACE2	angiotensin-converting enzyme 2
AMPK	AMP-activated protein kinase
ATF6	activating transcription factor 6
ATG	autophagy-related gene
BBH	berberine hydrochloride
BiP	immunoglobulin heavy chain binding protein (also known as GRP78)
CMA	chaperone-mediated autophagy
CQ	chloroquine
ER	endoplasmic reticulum
ERS	endoplasmic reticulum stress
GRP78	78 kDa glucose-regulated protein
HO-1	heme oxygenase-1
IEC	intestinal epithelial cell
IFN	interferon
IRE1	inositol-requiring enzyme 1
ISC	intestinal stem cell
ISG	interferon-stimulated gene
JAK	Janus kinase
KEAP1	Kelch-like ECH-associated protein 1
LIR	LC3-interacting region
MARCHF8	membrane associated ring-CH-type finger 8 (formerly MARCH8)
MAVS	mitochondrial antiviral signaling protein
mTOR	mechanistic target of rapamycin
NDP52	nuclear dot protein 52 (also known as CALCOCO2)
NRF2	nuclear factor erythroid 2-related factor 2
NSP	non-structural protein
PDCoV	porcine deltacoronavirus
PEDV	porcine epidemic diarrhea virus
PERK	PKR-like ER kinase
RIG-I	retinoic acid-inducible gene I
RLR	RIG-I-like receptor
ROS	reactive oxygen species
SAM	S-adenosyl-L-methionine
SARS-CoV-2	severe acute respiratory syndrome coronavirus 2
STAT	signal transducer and activator of transcription
TAK1	transforming growth factor-β-activated kinase 1
TBK1	TANK-binding kinase 1
TLR4	Toll-like receptor 4
TOM20	translocase of outer mitochondrial membrane 20
UBA	ubiquitin-associated domain
ULK1	UNC-51-like kinase 1
UPR	unfolded protein response
